# Revealing the role of microstructure architecture on strength and ductility of Ni microwires by *in-situ* synchrotron X-ray diffraction

**DOI:** 10.1038/s41598-018-36472-3

**Published:** 2019-01-11

**Authors:** Ravi raj purohit Purushottam raj purohit, Abhinav Arya, Girish Bojjawar, Maxime Pelerin, Steven Van Petegem, Henry Proudhon, Soham Mukherjee, Céline Gerard, Loïc Signor, Cristian Mocuta, Nicola Casati, Satyam Suwas, Atul H. Chokshi, Ludovic Thilly

**Affiliations:** 1 Institut Pprime, CNRS – ENSMA – Université de Poitiers, Département Physique et Mécanique des Matériaux, 86961 Futuroscope, France; 2 Department of Materials Engineering, Indian Institute of Science, Bangalore, 560 012 India; 3 MINES Paris Tech, Centre des Matériaux, CNRS UMR 7633, BP 87 91003 Evry Cedex, France; 4 Photons for Engineering and Manufacturing, Swiss Light Source, Paul Scherrer Institute, Villigen, Switzerland; 5 Synchrotron SOLEIL, L’orme des Merisiers, Saint Aubin – BP 48, Gif-sur-Yvette, 91192 France; 6 Laboratory for Synchrotron Radiation - Condensed Matter (LSC), Swiss Light Source, Paul Scherrer Institute, Villigen, Switzerland

## Abstract

Deformation mechanisms of cold drawn and electropolished nickel microwires are studied by performing *in-situ* monotonous and cyclic tensile tests under synchrotron radiation. X-ray diffraction tests allow probing elastic strains in the different grain families and establishing a link with the deformation mechanisms taking place within the microwires. The measurements were carried out on several microwires with diameters ranging from as-drawn 100 µm down to 40 µm thinned down by electropolishing. The as-drawn wires exhibit a core-shell microstructure with <111> fiber texture dominant in core and heterogeneous dual fiber texture <111> and <100> in the shell. Reduction of specimen size by electropolishing results in a higher yield stress and tensile strength along with reduced ductility. *In-situ* XRD analysis revealed that these differences are linked to the global variation in microstructure induced by shell removal with electropolishing, which in turn affects the load sharing abilities of grain families. This study thus proposes a new way to increase ductility and retain strength in nickel microwires across different diameters by tuning the microstructure architecture.

## Introduction

Micro-Electro Mechanical Systems (MEMS) are becoming more widely used with the scale reduction currently ongoing in the microelectronics industry and the associated development of very small devices. The length scale of MEMS spans between a few tens and a few hundreds of micrometers. At such scale, mechanical properties of materials start to deviate from bulk-like behavior^1^. The associated size effects occur because of constraints in microstructure and/or specimen dimension, as demonstrated since the 1950s^2^ Size effects can be broadly classified into two types: intrinsic (associated with microstructure refinement) and extrinsic (associated with sample size reduction). The strength of polycrystalline metals increases with grain size reduction, according to the Hall-Petch relation^3,4^, a behavior that is sometimes followed by softening, or inverse Hall-Petch effect^5,6^, when the grain size is further reduced to few tens of nanometers. The strength of single crystalline materials increases with a decrease of specimen size in the micrometer regime^7–19^. In multi-crystalline metals (with only a few grains across thickness), the specimen size (t) and the grain size (d) result in coupled effects, which in turn control the strength of the material^20^. Literature related to experimental studies on interaction of extrinsic and intrinsic size effects usually deals with multi- or single crystalline samples, where the strongest coupling between the two size effects is generally observed. Some well-documented experiments on extrinsic size effect in metals in multi-micrometer regime can be found in refs^9,20–27^, as well as at micron and sub-micron scale^28–30^. Some of these studies considered possible interaction between grain size and specimen size reduction^20,22,25–31^, whereas others did not^9,21,23,24^.

Literature in the field of multi-crystalline samples is contradictory with two prominent observations: the so-called “smaller is stronger”^31–33^ and “smaller is weaker”^22,29,34,35^. A systematic classification of these effects is provided by Janssen *et al*.^36^, who report possible competition between microstructural constraints, strain gradients, constraints on the surface layer and statistical effects. Experimental observations by other groups on face-centered cubic (FCC) metal thin films^22,37^ indicate a “smaller is weaker” effect. Lederer *et al*.^38^ worked on pure aluminium foils at elevated temperatures that exhibited the same “smaller is stronger” effect as in^31^, but the observed strengthening with decrease in thickness was explained by the formation of a surface oxide layer that limits dislocation escape. Another study on cold rolled thin aluminium sheets by Janssen *et al*.^36^ reported that the flow stress increases strongly when t/d is less than a critical value of about 3. In this study, no oxide layer was observed and the observed strengthening was attributed to the presence of grain boundaries parallel to the specimen surface that induce constraints on the grains during the rotation associated to plasticity. A more recent study on cold rolled and annealed Ni sheets revealed a complex behavior with the strength increasing and then decreasing when the t/d ratio is less than 5^20^. In microwires with few grains across thickness, the effect of specimen diameter was reported to be significant on the properties of the material: in the case of t/d smaller than five, microwires behave as thin plates and exhibit a “smaller is weaker” trend^39–43^.

Severe plastic deformation (SPD) processing techniques applied to metals result in ultra-fine grain (UFG) microstructure with grain size ranging between ~100 nm and 1 μm, that can exhibit high strength and high ductility. Most SPD-related studies deal with intrinsic size effects and possible impact of microstructure architecture such as bimodal grain size distribution^44,45^. In the present study, we focus on nickel (Ni) processed by severe drawing to obtain microwires: the literature on Ni and size effects is rather extended but one can refer to the work of Greer *et al*.^13^, Rinaldi *et al*.^24^, Frick *et al*.^46^ and Sun *et al*.^47^ on nanocrystalline (NC) Ni to gain insight into the diversity of observed intrinsic size effects. Regarding extrinsic size effects, studies on SPD-produced and annealed Ni thin films^20,29,30^, SPD-produced and annealed Ni microwires^21,48–50^ indicate different trends in mechanical properties with reduction of specimen size. For example, annealed thin sheets with polycrystalline microstructure (with more than 20 grains across thickness) do not exhibit dependence with respect to specimen thickness, indicating that extrinsic size effect comes into play only when the number of grains across thickness is reduced below a critical value of four^20,29,30^.

Recent tensile tests on commercially available (from Alfa Aesar) Ni microwires by Warthi *et al*.^48^ demonstrated a significant extrinsic size effect when the wires are thinned down to 20 µm by electropolishing from an initial cold drawn 100 µm wire. The tensile strength of thinnest electropolished wires was seen to approach the theoretical strength and to be much higher than nanocrystalline Ni. Similar tests (Agepati *et al*.^49^) on a second batch of Ni microwires from the same commercial source yielded a huge scatter in tensile strength. As a summary, these studies have shown that cold drawn Ni microwires seem to be sensitive to extrinsic size effect, with increase in tensile strength and reduction of ductility when reducing their diameter by electropolishing, i.e. without changing their internal microstructure. It should be noted that any possible surface features and electropolishing artefacts have been ruled out as possible origin of observed size effect^48,49^.

The objective of this work is thus to understand strengthening and reduction of ductility in nickel microwires with reduction in diameter via high-energy synchrotron X-ray diffraction (XRD). Tensile tests on Ni microwires are performed in combination with XRD to derive the deformation mechanisms taking place in the different grain families. These mechanisms are discussed in view of the initially observed microstructure (grain size and crystallographic micro-texture) and the effect of diameter change by electropolishing. From these results, guidelines are proposed to tailor the strength and ductility of Ni microwires, these considerations being general enough to be extended to other FCC metals.

## Methods

### Sample preparation and characterization

The material used in this study is a high purity (~99.994%) Ni microwire of ~100 µm diameter obtained from Alfa Aesar by cold drawing (details on its previous thermo-mechanical history was not made available). Main impurities present in the Ni microwire as provided by Alfa Aesar are (in wt. ppm): Fe = 10, C = 15, Cu = 0.56, Si = 0.12, O = 20, S < 1, N < 1, H = 1. A discussion on the impurity content, intermediate heat treatments and their effect on the mechanical properties is not in the scope of current study. For more information, the reader is referred to^51–53^.

The microwire cross-sectional surface was polished by dual-beam focused ion beam (FIB) workstation using FEI Nanolab Helios G3 CX with a very low current before characterizing the microstructure by electron back-scatter diffraction (EBSD). The EBSD acquisition was done with 50 nm step size to have a good resolution of the submicron grains. Analysis was carried out by TSL OIM^TM^ software to compute the micro-texture, grain size distribution and grain boundary misorientation distribution. A tolerance angle of 2° and minimum grain size of 25 pixels were chosen for the cleanup of EBSD data. Several EBSD cross-sectional maps of Ni microwire were obtained on entire wire sections taken few meters apart in length to ensure the representativeness of grain size and texture distribution.

From the initial cold drawn Ni microwire labelled as AA100, with a diameter of 103 µm ± 1 µm, several wires of 70 µm, 50 µm, 40 µm diameters were produced by electropolishing, labelled respectively as EP70 (diameter: 69 µm ± 1.5 µm), EP50 (diameter: 49 µm ± 1.5 µm) and EP40 (diameter: 40 µm ± 2.5 µm). Note that the error bar (standard deviation) on diameter reflects the difference in diameter between the different samples and not the diameter variation along one single wire. The electropolishing was carried out with an electrolyte having a volumetric ratio 9:1 of ethanol to perchloric acid under a potential of 3.3 V, at a temperature of 250 K. The wire was mounted horizontally in the electrolyte to minimize the impact of temperature gradient and stirred to obtain a uniform diameter. The surface of the electropolished wires were checked and measured using JEOL Field Emission Gun (FEG) - Scanning Electron Microscope (SEM). Only specimens with a smooth surface and uniform diameter having a variation of ±1.5 µm or less along the length were selected for the *in-situ* tests.

### *In-situ* experimental setup and procedure

**Figure 1:**
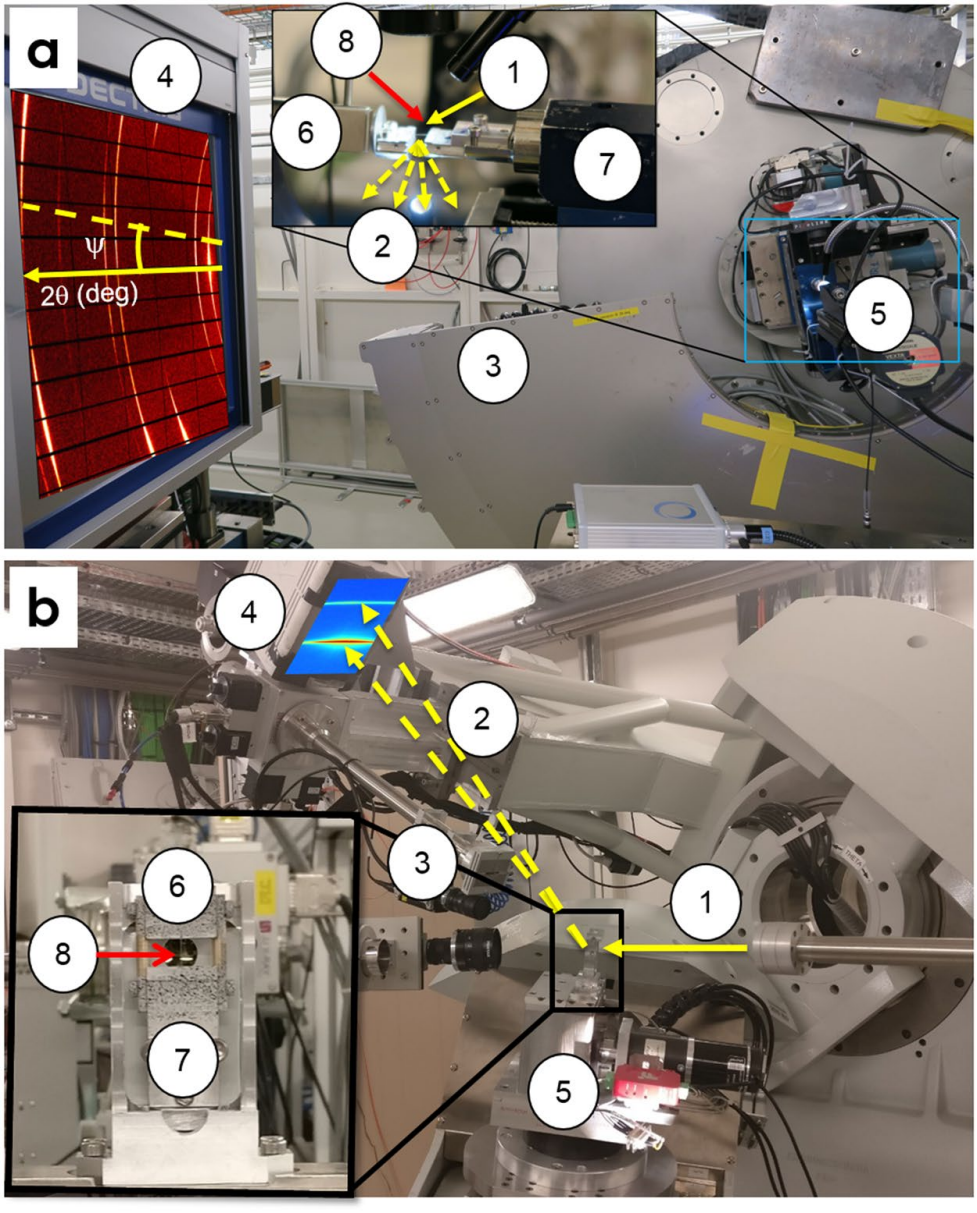
High energy *in-situ* XRD setup at (**a**) MS beamline (26 keV), SLS, Switzerland (**b**) DiffAbs (19 keV), SOLEIL, France. The setup includes (1) incident X-ray beam, (2) diffracted X-ray beam, (3) 1D XRD detector, (4) 2D XRD detector (a: PILATUS 6 M, b: XPAD S140), (5) Micro Tensile Machine (MTM), (6) fixed grip of MTM, (7) moving grip of MTM, (8) Ni microwire mounted on specific holder.

*In-situ* tests were performed under high-energy synchrotron XRD at room temperature. Figure 1 shows the different setups used at Materials Science (MS) beamline of Swiss Light Source, Switzerland, and DiffAbs beamline of SOLEIL, France. The major differences between the two beamlines are: the incident X-ray beam energy (MS: 26 keV; DiffAbs: 19 keV), the type of detectors (MS: 1D microstrip detector MYTHEN^54^ and area detector Pilatus 6M^55^; DiffAbs: area detector XPAD S140^56^), the detector acquisition time (MS: 10–30 seconds; DiffAbs: 1–3 seconds) and beam dimensions (MS: 200 µm high x 600 µm wide, DiffAbs: 250 µm high x 250 µm wide). Two specifically designed micro-tensile machines (MTMs) (MS^57^, DiffAbs^58^) were used to perform monotonous tensile, load-unload, stress-drop and strain rate jump tests. Stress drop and strain rate jump tests were only performed on the AA100 wire at MS beamline.

A specific sample holder compatible with the two different MTM at MS and DiffAbs beamlines was designed to ease the sample handling and to minimize the sample vertical movement during the tensile test under the X-ray beam (refer to Supplementary Fig. S1). Supplementary methods describe the procedure for mounting the microwires in the specific holder and how the wire alignment was further checked before *in-situ* experiments.

Once the sample holder (and sample) was mounted on the MTM, the incident X-ray beam was aligned to the center of the microwire.

For the *in-situ* tests, only the evolution of <111> and <100> oriented grains (also called “main grain families” as presented later) was followed (main grain families as presented later). The different diffraction geometries used at MS and Diffabs beamlines are described in details in Supplementary methods. For the sake of clarity only axial (111) and (200) reflections will be presented here and compared between different wire diameters.

### Analysis and interpretation of XRD data

For the patterns (axial reflections) recorded with 2D detector, where a part of the Debye-Scherrer rings is recorded, a preliminary process of detector calibration by a standard material (Lanthanum hexaboride powder) and flat field recording is carried out for later correction with PyFaI software^59^. In order to evaluate the peaks profiles, the rings are processed by caking along angle ψ (refer to Fig. 1a). Because of preferred orientation, regions of high intensity appear along the rings and the caking angle is defined to include the high intensity segments of the rings (here Δψ = 5°). The integration along ψ yields 1D diffraction pattern with respect to 2θ.

The 1D pattern is then fitted with a split Pearson VII function to resolve parameters related to the shape of the peak, the peak position (2θ) and the peak width or Full Width Half Maximum (FWHM) (from equations (1) and (2)). Each peak is evaluated separately and a single peak is described by:

1 $$I(2\theta )={I}_{max}\times {[1+({2}^{\frac{1}{{M}_{r}}}-1)\times {({10}^{-\alpha }+1)}^{2}\times (\frac{\theta -{\theta }_{hkl}}{FWHM})]}^{-{M}_{r}},\,if\,\theta \ge {\theta }_{hkl},$$

2 $$I(2\theta )={I}_{max}\times {[1+({2}^{\frac{1}{{M}_{l}}}-1)\times {({10}^{\alpha }+1)}^{2}\times (\frac{\theta -{\theta }_{hkl}}{FWHM})]}^{-{M}_{l}},if\,\theta  < {\theta }_{hkl},$$

where θ is the diffraction angle, θ_hkl_ is the peak maximum angle of the corresponding (hkl) reflection, I_max_ the maximum peak intensity or peak height, FWHM the peak width, α the logarithmic asymmetry parameter, M_r_ and M_l_ are the right and left decay component, the value of which gives Pearson VII a Gaussian or Lorentzian character. A detailed information regarding the peak fitting analysis procedure can be found in paper^57^.

X-ray diffraction probes the changes in the lattice spacing during deformation. Such lattice changes or lattice strain can be followed for selected grains to get the lattice strain of the material in that particular < hkl > direction. The lattice strain ${\varepsilon }_{hkl}\,$is represented in microstrain units by the following relation:

3 $${\varepsilon }_{hkl}=-\,\cot ({\theta }_{hkl}){\rm{\Delta }}{\theta }_{hkl}\times {10}^{6}$$

where ${\rm{\Delta }}{\theta }_{hkl}$ is the difference of peak position calculated from the current ${\theta }_{hkl}$ and the position before loading.

When plotting lattice strain with respect to applied stress, the deflection of lattice strain from linearity indicates the onset of plastic regime: a negative deviation from linearity of a particular (hkl) reflection is related to the grains that are deforming plastically, whereas a positive deviation from linearity is indicative of elastic load takeover or ‘load-transfer’ from the plastic grains to the still elastic grains^60,61^.

Further information can be obtained from the FWHM analysis. The FWHM here is presented in terms of scattering vector ‘s’, calculated with the following relation:

4 $$\delta {s}_{hkl}=\frac{2\times \,\cos \,{\theta }_{hkl}\times FWH{M}_{hkl}}{\lambda }$$

where FWHM_hkl_ is the measured FWHM in radians and λ is the wavelength of X-rays used.

The micrometer-size of grains in Ni micro-wires does not significantly contribute to diffraction peak broadening. Hence, the FWHM in initial state can result from intergranular strains due to difference in the average strains of different grains and/or intragranular strains due to the presence of dislocations within grains. During *in-situ* deformation, when dislocation density increases, the FWHM is expected to increase. The analysis of lattice strain and evolution of FWHM during deformation thus provides the possibility to follow the evolution of microstructure (including dislocation storage) and to detect elastic-plastic transition within different grain families^60,62^.

The diffraction data collected here exhibit a relatively good quality with reduced background and noise levels so, in principle, the peak broadening (FWHM) could be quantitatively analyzed in terms of inhomogeneous strains, as done in numerous reference studies^63–65^. However, since each major grain family (<111> and <100> oriented grain families) is represented by a single reflection ((111) and (200) respectively), it was decided to restrict to qualitative analysis of the FWHM evolution. Moreover, in the current study, the *in-situ* XRD data interpretation is based on the relative changes of the measured values and not the absolute values, hence is not addressing the topic of residual stresses.

### Monotonous tensile and load-unload tests

**Figure 2:**
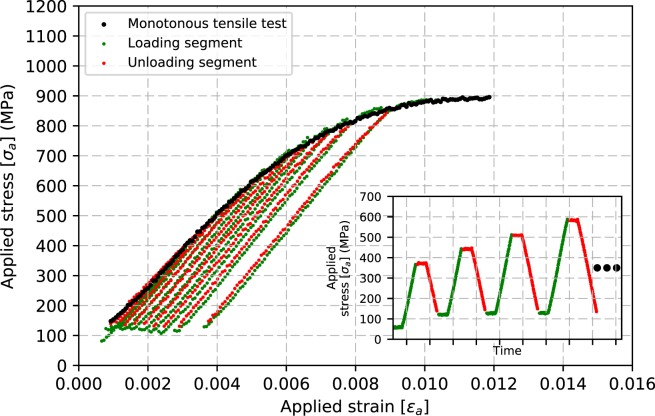
Monotonous and cyclic load-unload tensile curves for AA100 microwires. The inset shows the stress-controlled load-unload test. A cycle here refers to one load (green) and one unload (red) segments, each cycle ending with tensile stress of 100 MPa to avoid bending and movement of wire in the beam.

Monotonous and cyclic tensile tests were carried out at both beamlines at room temperature. The data were collected at DiffAbs with faster acquisition and hence, more data points were obtained. For the sake of simplicity, only the DiffAbs data will be discussed in this section but results obtained at MS have led to similar conclusions. The fast acquisition time of the detector and the high X-ray flux made it possible to conduct continuous uniaxial tensile and cyclic tests on AA100, EP70, EP50 and EP40 wires at a constant strain rate of$\,8.3\times {10}^{-6}{s}^{-1}$. At the beginning of the test, samples were pre-strained to align them with respect to the X-ray beam, this pre-straining ensures minimal displacement of the volume being probed during the test (hence the presented stress-strain curve has initial values of stress and strain higher than zero). Figure 2 presents in a superimposed way the monotonous true stress-true strain and cyclic (load-unload) true stress-true strain plots for two similar AA100 wires, each data point corresponding to a recorded XRD pattern. It should be noted that each MTM compliance contribution has not been removed but is expected to become negligible for smaller diameter wires, since the applied forces are becoming very small. The engineering stress is calculated from the load over the initial cross-section of the wire, while the engineering strain is calculated from the grips displacement measured over the initial gauge length of the microwire. The stress-strain has then been converted to applied stress-strain (true stress-strain) using the standard equations. Also, only data points until the onset of necking are considered. *In-situ* tests were performed at least three times for monotonous tensile tests and twice for cyclic tests for each wire dimension to ensure reproducibility of the results.

### Stress-drop and strain rate jump test

Due to the extended close-to-zero hardening regime observed in AA100 wires, the stress drop and strain rate sensitivity tests were conducted only for the AA100 wires and at MS beamline. Supplementary methods describe how these tests were conducted and analyzed. The stress-drop tests provide insights into the deformation mechanism responsible for the changes in diffraction peak width (FWHM) in the macro-plastic regime by suppressing dislocation slip: as a reference, one can refer to stress drop tests performed on electrodeposited NC Ni^47^. From strain rate jump tests, the strain rate sensitivity index, m, can be calculated: higher values of m (>0.3) are considered as footprint of superplastic behavior. A strain rate sensitivity index of nearly zero indicates rate-independent plastic flow. A detailed study on strain rate sensitivity for micro- (>1 µm), ultrafine (100–1000 nm) and nano-grain (<100 nm) Ni can be found in refs^66,67^.

## Results

### Microstructure of Ni microwires

**Figure 3:**
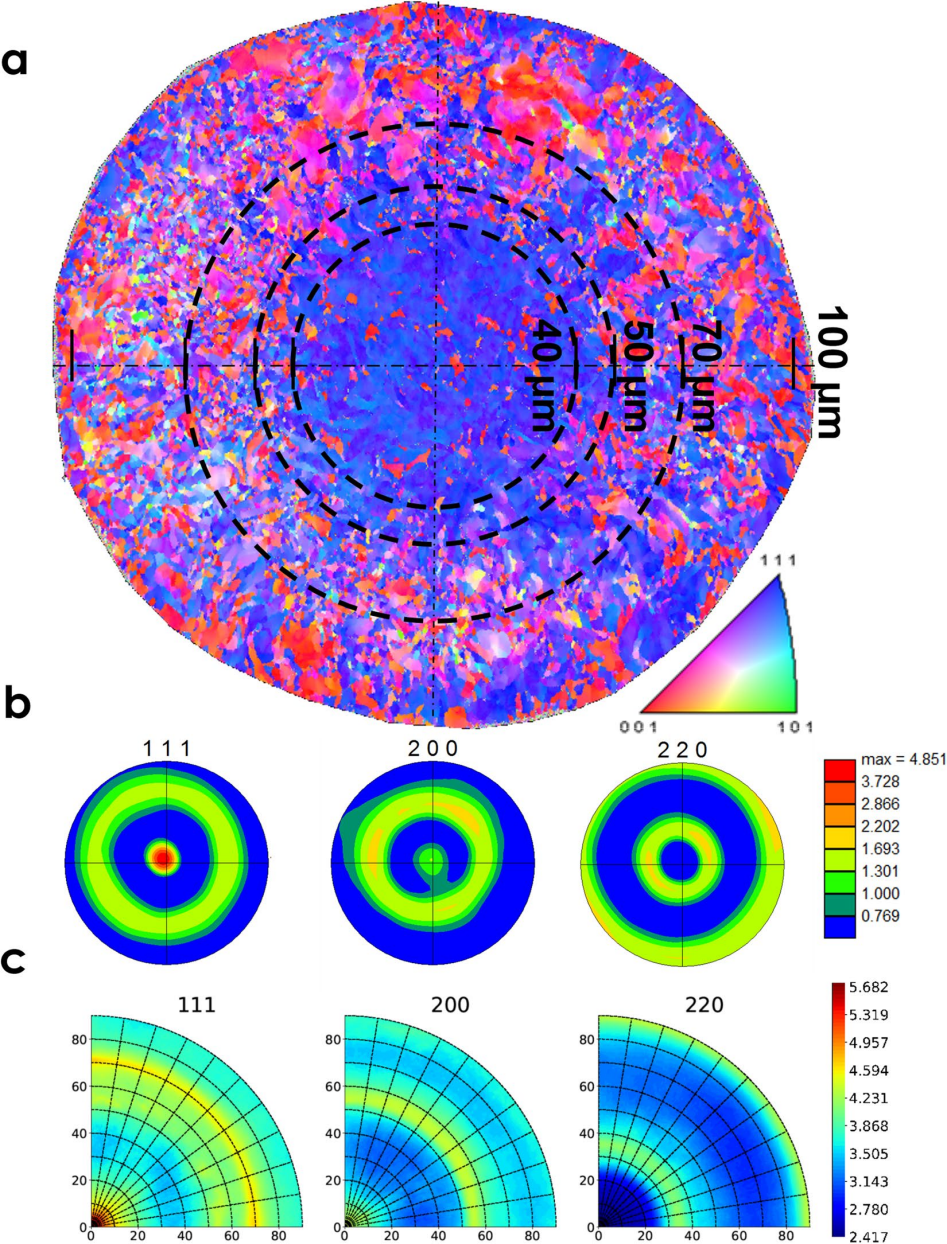
Texture analysis of 100 µm Ni microwire. (**a**) Cross-sectional EBSD map (IPF relative to the wire axis); the black dashed circles correspond to the different diameters obtained by electropolishing. (**b**) (111), (200), (220) pole figures from cross-sectional EBSD. (**c**) (111), (200), (220) pole figures along wire axis from high-energy X-ray diffraction.

An example of cross-sectional EBSD map as well as pole figures obtained using both EBSD and high energy X-ray diffraction is shown in Fig. 3: according to the inverse pole figure (IPF) map shown in Fig. 3(a), it can be seen that the micro-texture of 100 µm wire exhibits a ‘core-shell’ type microstructure, that will be commented below. The microstructure analysis from several cross-sectional EBSD maps revealed that the wire is composed of equiaxed grains with size ranging from 0.4 µm to few micrometers: the core of the microwire has a rather uniform grain size up to 8 µm while shell exhibits a non-uniform distribution with a higher fraction of grains in the submicron size regime. The grains have an average cross-section aspect ratio of 0.8–1.0 (in core and shell) and are strongly elongated in the wire axis direction with an average length of 50 µm in the core and 25 µm in the shell, resulting in a very high longitudinal aspect ratio in the range of 10–30. Grain boundary analysis indicates that core of the microwire has a slightly higher fraction of low angle grain boundaries, whereas the shell region exhibits higher fraction of high angle grain boundaries. Both core and shell have very low fraction of Σ3 boundaries. As mentioned above, the texture components in the wire have a strong correlation with spatial location along the wire cross-section: the core has a strong <111> fiber texture (marked by the innermost circle of 20 µm radius), while the shell exhibits a dual texture with majority of grains having <111> and <100> orientation, as well as several intermediate orientations. The EBSD pole figures from Fig. 3(b) and XRD pole figures from Fig. 3(c) indicate double fiber textures with grain fiber axis being <111> and <100>, coinciding with wire axis. The angular width of these texture components gives the degree of scatter of the texture. The EBSD cross-sectional map and the associated pole figures are representative of the wire microstructure as shown by the good agreement with macroscopic XRD pole figures.

The expected microstructure of the electropolished diameters is illustrated in Fig. 3(a) by the series of dashed circles. Homogeneous reduction of wire diameter by electropolishing should lead to a decrease in the shell volume fraction, the smallest diameter achieved being 40 µm diameter (or less), where the shell is expected to be completely removed.

**Figure 4:**
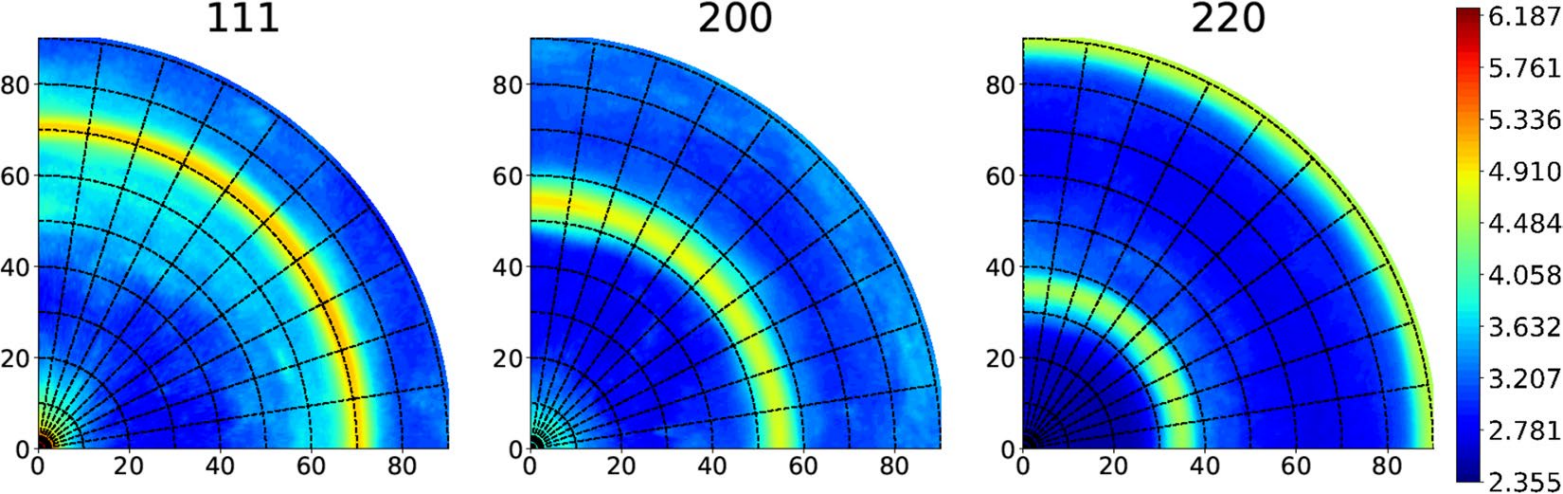
(111), (200), (220) pole figures of 40 µm microwire along wire axis from high-energy X-ray diffraction.

The XRD pole figures of an EP40 wire is shown in Fig. 4. The absence of intermediate orientations can be observed from the (111) and (200) macroscopic XRD pole figures as well as a strong <111> fiber texture with a very minor <100> component. Comparing the macroscopic XRD pole figures of AA100 from Fig. 3(c) with that of EP40 from Fig. 4, it is clear that with electropolishing the heterogeneous shell volume fraction is removed and the scatter of texture along the fiber axis is also markedly reduced.

Based on the maximum angular spread of the (111) pole, a 10° integration window is considered for the calculation of volume fractions of macroscopic texture components. The analysis of AA100 wire’s pole figures indicates 75% vol. of <111> and 25% vol. of <100> while for the EP40 wire the volume fraction of <111> component was 95% and a mere 5% for <100>. For the intermediate diameters EP70 and EP50, XRD radial texture analysis was carried out for calculation of volume fraction of <111> and <100> texture components and is shown in Table 1. The gradual decrease in volume fraction of <100> component with electropolishing is in line with the micro-texture observations by EBSD. AA100 wire has more inhomogeneous microstructure because of the presence of distinct core and shell, whereas for the EP40 wire the inhomogeneity is removed by electropolishing the shell. This difference in micro- and macro-texture introduced as a result of electropolishing is expected to have a major influence on the strength and ductility of the microwires.

**Table 1:** Characteristics of microwires (a minimum of three wires have been deformed under X-rays: error bars correspond to the data scatter).

Sample ID	Vol. fraction of <111> grains	Vol. fraction of <100> grains	Yield stress (MPa)	Tensile strength (MPa)	Uniform elongation (%)
AA100	0.75	0.25	820 ± 7	885 ± 12	1.2 ± 0.17
EP70	0.83	0.17	850 ± 26	895 ± 30	0.82 ± 0.09
EP50	0.90	0.10	915 ± 90	940 ± 119	0.61 ± 0.07
EP40	0.95	0.05	1050 ± 56	1050 ± 56	0.54 ± 0.08

### Effect of sample size on tensile strength and ductility

**Figure 5:**
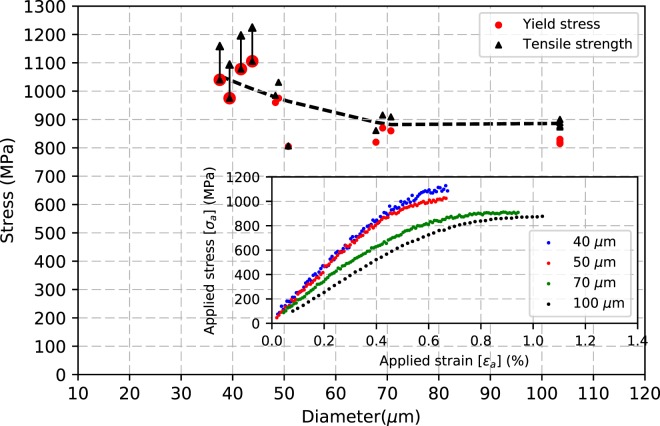
Effect of diameter on yield stress and tensile strength of Ni microwires. Inset corresponds to typical tensile stress-strain curves of AA100, EP70, EP50 and EP40 microwires. The black dashed line serves as a guide to the eye (tensile strength data).

As shown in Fig. 5, the yield stress (definition of yield stress used in the present study is given in the next section) and tensile strength of the microwire increase with decreasing wire diameter by electropolishing. A noticeable increase in strength and decrease in uniform elongation is seen at 50 µm diameter (refer to Fig. 5 inset). The difference in the yield stress and tensile strength decreases with decrease in wire diameter and overlaps for 40 µm wires. Monotonous tensile tests on AA100 wires show an average elongation of 1.2% before necking, whereas for the EP40 wires such elongation is not seen at all as the wire necks soon after yielding. In other words, EP40 do not exhibit any hardening plateau, in contrast to AA100. These differences are attributed to the change of representative microstructure induced by electropolishing.

From Fig. 5 (inset), the slope of applied stress-strain curves in elastic regime increases with reduction in wire diameter. We recall that the directional Young’s modulus (calculated with respect to single crystal elastic constant given in^68^) of Ni ranges from 131 GPa for loading in <100> direction to 288 GPa for <111> direction. The summary on volume fraction and mechanical properties of the microwires are presented in Table 1.

### Evolution of lattice strain and peak broadening during the tensile and cyclic test

**Figure 6:**
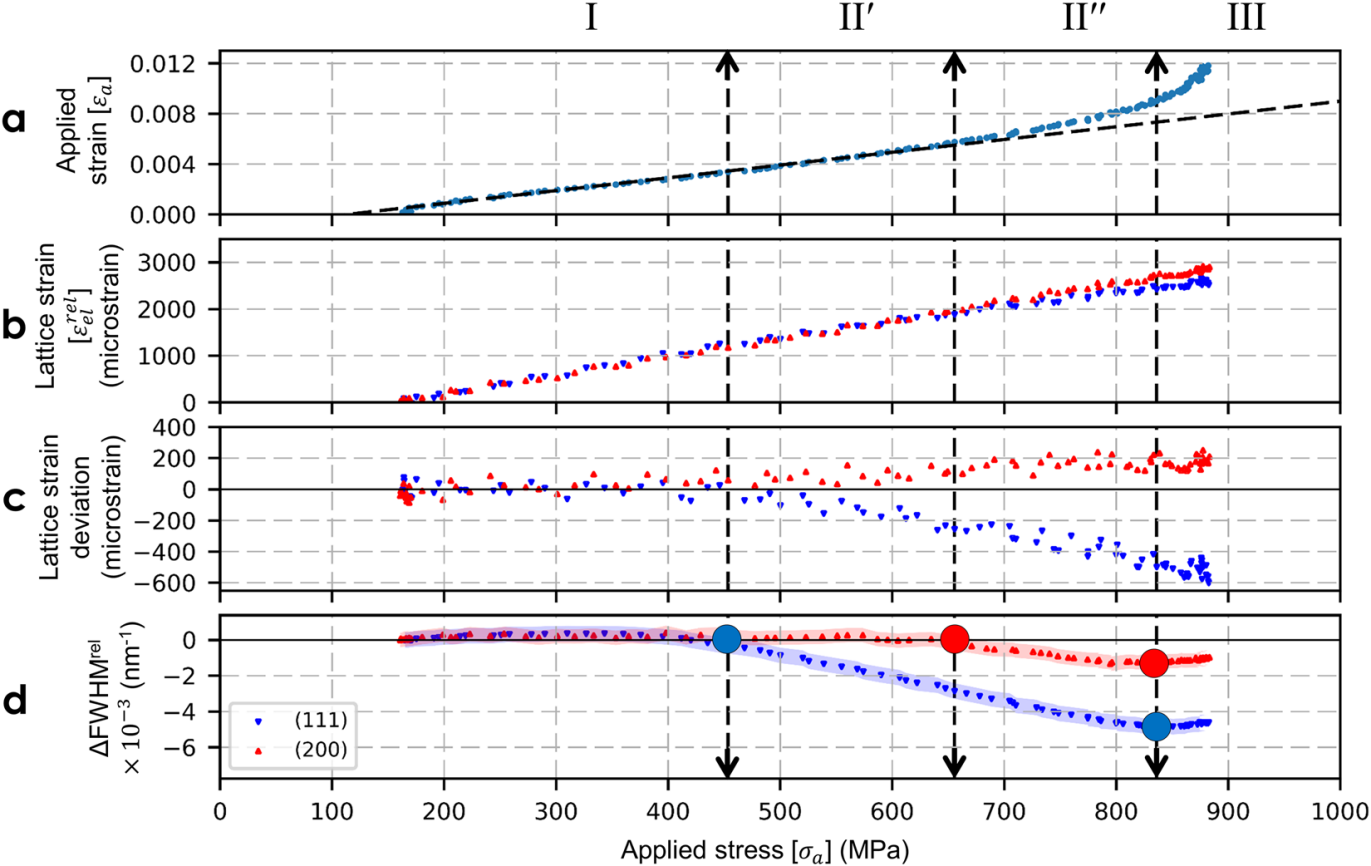
XRD peak analysis of AA100 wire (data points before the onset of necking are only presented). Top plot (**a**) corresponds to continuous tensile strain-stress data, middle plots (**b,c**) corresponds to the evolution of axial (111) and (200) elastic lattice strains and their deviation from linearity versus applied stress (linear fits are carried out for data points below 450 MPa), bottom plot (**d**) corresponds to the evolution of the (111) and (200) Full Width Half Maximum (FWHM) versus applied stress, the shaded zones represent the error bar. The vertical dashed lines in the plots represent different stress levels corresponding to the evolution of FWHM (see text).

Figure 6 shows XRD peak analysis of AA100 wire. The top plot shows the true strain- true stress curve (reader’s attention is drawn to this inverted representation compared to traditional stress-strain curve), where each data point corresponds to a recorded diffraction pattern. The two middle plots show the axial lattice strains derived from (111) and (200) axial reflections and their deviation from linearity, as a function of applied stress. The bottom plot presents the variations of FWHM, for (111) and (200) axial reflections, with respect to applied stress. Analysis of lattice strain deviation from linearity indicates that, up to an applied stress of about 450 MPa (marked by the first vertical dash line, at corresponding applied strain of ~0.3%), both <111> and <100> grain families deform elastically. Then (111) axial strain exhibits a negative slope change that can be understood as the footprint of first plastic events, inducing a load transfer from <111> grains to <100> grains as confirmed by the positive slope change in (200) axial reflection. Above 450 MPa, i.e. during the early stages of plasticity (or micro-plastic regime), a decrease in FWHM for the <111> grain family is seen. At higher stress of about 650 MPa (marked by the second vertical dash line at corresponding applied strain of ~0.55%), a decrease in FWHM is also seen for <100> grain family. Above 830 MPa (third vertical dash line at a corresponding applied strain of 0.9%), the macro-plastic regime is characterized by an increase in FWHM in both grain families.

Hence, three distinct regions for the AA100 wire can be identified based on the evolution of FWHM with respect to applied stress. Regime I is defined as the range where there is minimal or no changes in the FWHM behavior with applied stress for both (111) and (200) reflections: regime I corresponds to the elastic regime. The first vertical line indicates the end of regime I and the start of regime II associated first with the decrease of FWHM for (111) reflection (sub-regime II′) and second with the decrease of FWHM for (200) reflection (sub-regime II″), at second vertical line. Sub-regime II′ marks the start of microstructural changes in <111> family of grains only [450–650 MPa]; sub-regime II″ is characterized by microstructural changes in both grain families and corresponds to the rounding of the macroscopic stress-strain curve [650–830 MPa]. Regime II (sum of sub-regimes II′ and II″) is thus defined as the stress range where the effective load transfer takes place between the grain families, marked by the reduction in FWHM and the deviation of lattice strains from linearity. Regime III starts with the increase in FWHM for both reflections (third vertical line) [>830 MPa]: this regime is associated to significant plastic deformation in all major grain families and a macroscopic yield criterion can be defined based on the stress at which regime III starts. This criterion has been applied to obtain the data presented in Fig. 5 and Table 1.

**Figure 7:**
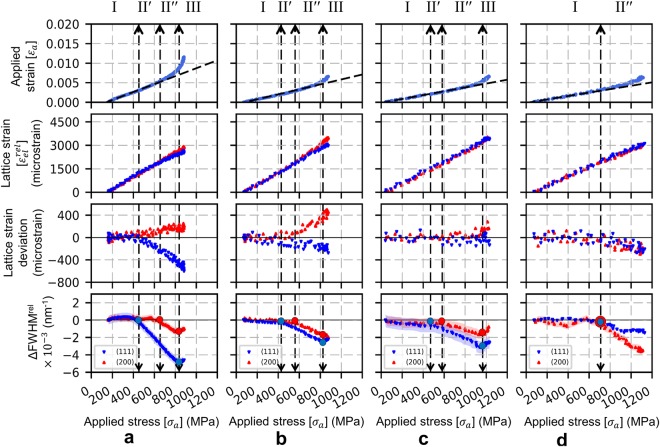
XRD peak analysis of Ni microwires, (**a**) AA100, (**b**) EP70, (**c**) EP50, (**d**) EP40. Top plots correspond to continuous tensile strain-stress data, middle plots corresponds to the evolution of axial (111) and (200) elastic lattice strains and their deviation from linearity versus applied stress (linear fits are carried out for data points below 450 MPa), bottom plots corresponds to the evolution of the (111) and (200) Full Width Half Maximum (FWHM) versus applied stress, the shaded zones represent the error bar. The vertical dashed lines in the plots represent different stress level corresponding to the evolution of FWHM (see text).

Figure 7 shows the XRD peak analysis of AA100, EP70, EP50, EP40 wires. Regime I (elastic behavior in all grains) extends to the same stress value (about 450 MPa) for AA100, EP70 and EP50 microwires but extends to a larger value (about 700 MPa) for EP40 microwires. With reduction in wire diameter, interesting features can be observed during regime II: first, regarding the lattice strains deviation from linearity, the (200) lattice strain exhibits a positive slope change in AA100 microwire and a negative slope change in EP40 microwire. As explained previously for the AA100 microwires, this suggests a strong load transfer between <111> grains and <100> grains in the presence of ‘core-shell’ microstructure while this load transfer disappears for the EP40 microwires, with only ‘core’ microstructure. Second, regarding the FWHM behavior, the extension of Regime II′ decreases with decreasing wire diameter and cannot be observed for the EP40 microwires: in the latter case, only regime II″ is seen, with decrease of FWHM for both (111) and (200) reflections. Regime III, associated to FWHM increase for all reflections (and hence macro-plasticity) starts at similar applied stress for AA100 and EP70 microwires (about 830 MPa), shifts to about 950 MPa for EP50 microwires and is not seen for EP40 microwires. The Regime III is not reached in EP40 because of early onset of strain localization resulting in failure before reaching homogeneous deformation. Hence the yield stress and tensile strength of the wire overlap in Fig. 5, indicating that the current definition of yield stress defines the yield as the lower limit for the EP40 wires and is expected to increase had the wire not failed sooner due to strain localization.

**Figure 8:**
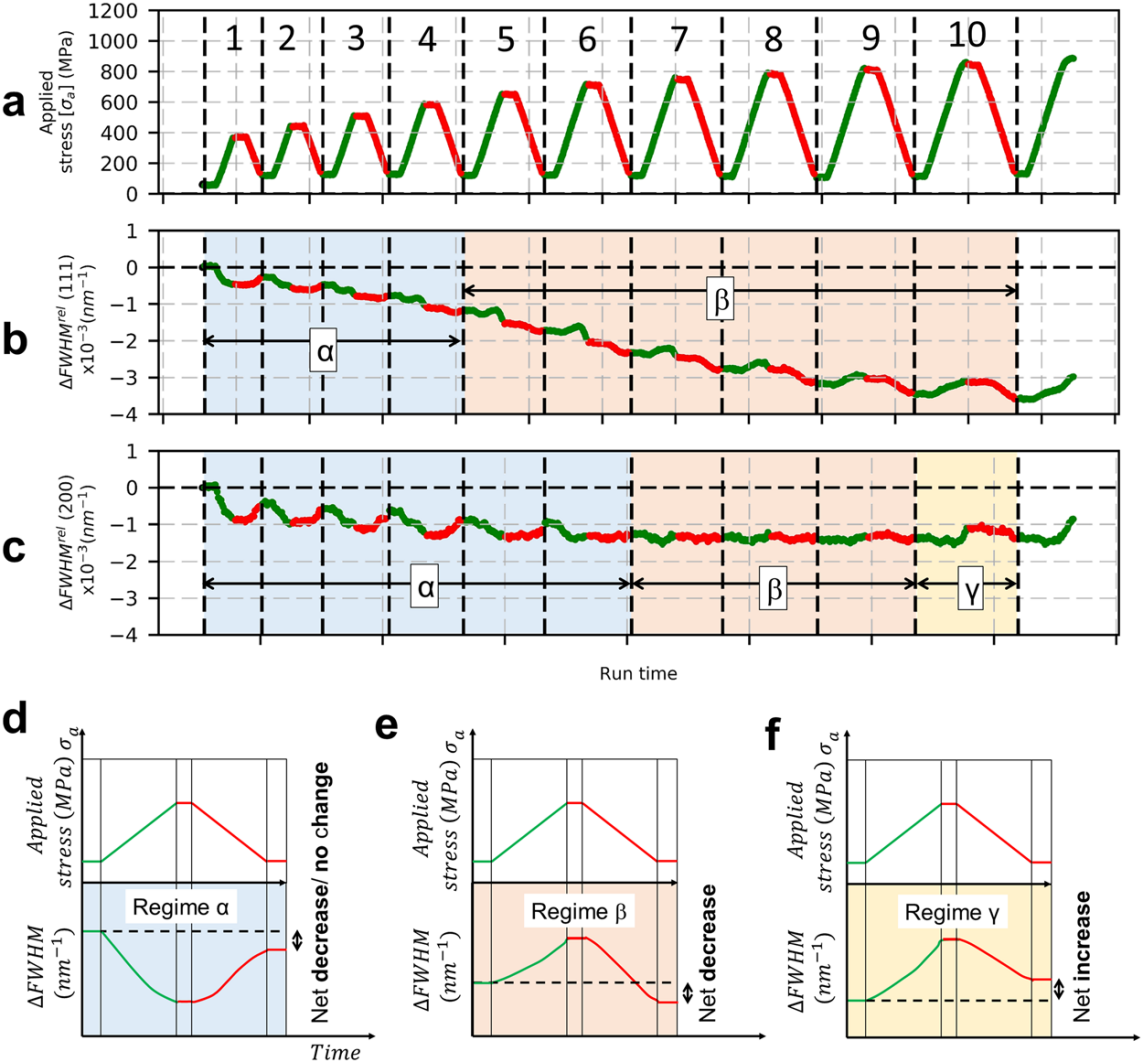
Load-unload test on AA100 microwire. (**a**) stress with respect to time, (**b**) evolution of FWHM of (111) reflection with time. (**c**) evolution of FWHM of (200) reflection with time. (**d**) schematics of evolution of FWHM in Regime α, (**e**) schematics of evolution of FWHM in Regime β, (**f**) schematics of evolution of FWHM in Regime γ.

The evolution of FWHM is investigated further by carrying out load-unload cycles. For the sake of simplicity only two cases (AA100 and EP40) of cyclic results are discussed here. Figure 8(a) presents the load-unload cycles of AA100 and the evolution of FWHM for (111) and (200) reflections with respect to time. The corresponding true stress-true strain curve is shown in Fig. 2. Distinct regimes have been categorized based on the evolution of FWHM with load-unload cycles and labelled as α, β and γ, refer to Fig. 8(d,e,f).

In the case of AA100 wires, regime α, which marks the net decrease in FWHM is seen for both the (111) and (200) reflections. For (111) reflection the regime can be seen up to four load-unload cycles (maximum applied stress of 600 MPa), whereas it is seen up to six load-unload cycles in the case of (200) reflection (maximum applied stress of 700 MPa). It should be also mentioned that the cyclic test of AA100 wire was started at a stress level of 50–100 MPa and each unloaded state corresponds to an applied stress of 100 MPa.

Regime β is characterized by an increase in ΔFWHM upon loading and a decrease upon unloading, such that there is a net decrease of ΔFWHM at the end of the cycle: it is observed for both the (111) and (200) reflections. For (111) reflection the regime seems to appear towards the end of cycle 4 and extends until the end of the test (maximum applied stress of 900 MPa), whereas for the (200) reflection, this regime starts at cycle 7 and is seen until the end of cycle 9 (maximum applied stress of 800 MPa). The magnitude of the net decrease in ΔFWHM depends on reflections, i.e. the (111) shows a continuous net decrease in ΔFWHM at the end of each load-unload cycle, whereas the (200) reflection rather shows a small variation. It should be mentioned that the transition from regime α to regime β is not very clear.

Regime γ, marks a regime where a net increase in ΔFWHM is seen. For the applied cycles (stopped before wire breaking), regime γ is only observed for the (200) reflection at the end of cycle 10.

**Figure 9:**
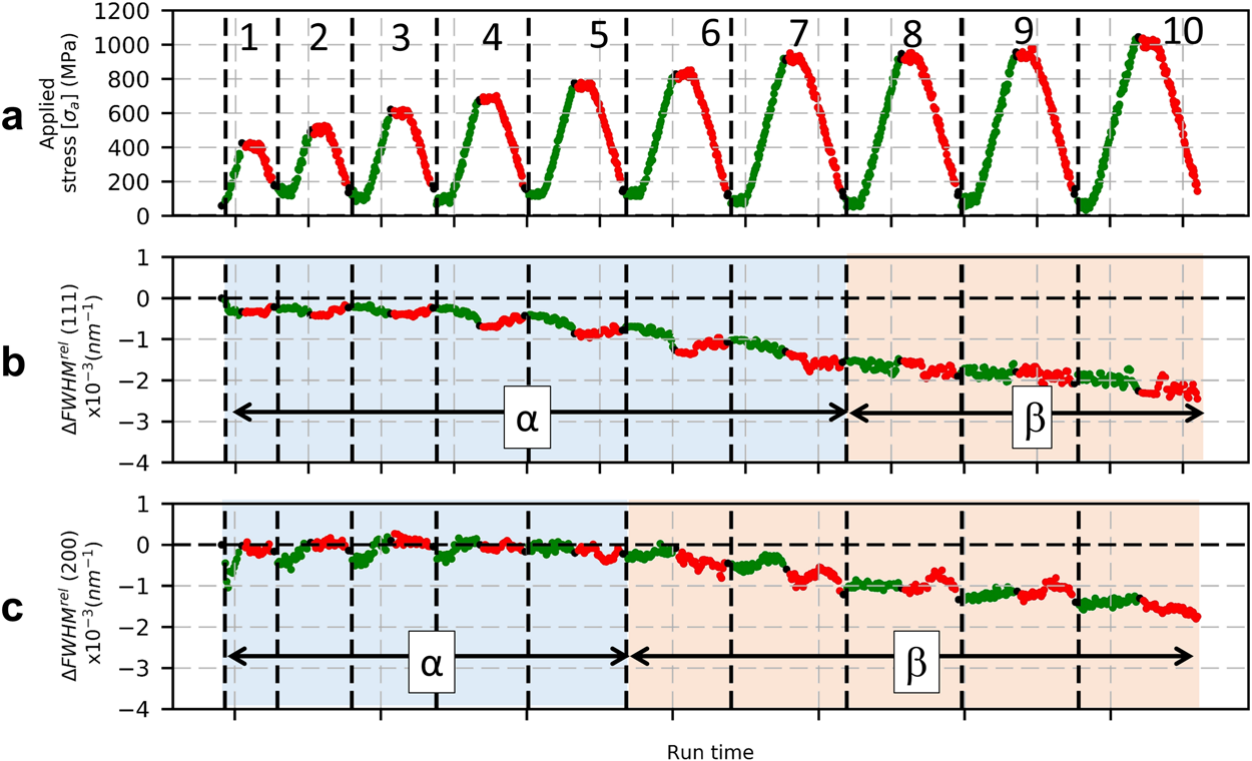
Load-unload test on EP40 wire. (**a**) stress with respect to time. (**b**) evolution of FWHM of (111) reflection with time. (**c**) evolution of FWHM of (200) reflection with time.

Figure 9 shows the load-unload test and evolution of FWHM for EP40 microwire. From the comparison between EP40 and AA100 wires, it can be observed that regime α for (111) reflection extends to higher applied stress of 900 MPa in EP40 wire and for the (200) reflection, it is seen until 800 MPa. The resolution of regime α is not so clear in the case of (200) reflection in EP40 wire, the noisy data can be explained by the very low diffracting volume fraction of <100> grains. In the case of regime β, the (200) reflection shows the start at 800 MPa, whereas it sets in later at 925 MPa for the (111) reflection. In the case of AA100 wire, the regime β is seen to occur first in (111) reflection at about 650 MPa and then the (200) reflection at about 750 MPa. Regime γ is not seen for both the (111) and (200) reflections in EP40 wire i.e., the wire necks before reaching a regime of homogeneous deformation. It can also be seen in the load-unload test that the FWHM of (111) and (200) reflections do not saturate, on contrary to what has been observed for AA100 wires.

## Discussion

### General considerations

EBSD maps obtained on cross-section of initial AA100 wires have revealed the presence of a specific core-shell microstructure, with major <111> fiber texture in the core and dual texture with <111> and <100> orientations in the shell. The homogeneous reduction of wire diameter by electropolishing should thus lead to a decrease in the shell volume fraction. In the case of the smallest diameter, with diameter of 40 µm diameter, the shell is expected to be completely removed.

The first impact of the reduction of the shell contribution by electropolishing is seen on the macroscopic mechanical tensile curves, with an increase in strength and decrease in uniform elongation that become more important below a diameter of 50 µm. The second impact is related to the slope of stress-strain curves in elastic regime that increases with reduction in wire diameter. We recall that Ni exhibit a rather strong elastic anisotropy with a directional Young’s modulus of 131 GPa in <100> direction and 288 GPa in <111> direction. Hence, the observed change in slope can be attributed to the increase in volume fraction of <111> fiber texture with gradual removal of the shell region.

These general considerations on the macroscopic mechanical properties are not sufficient to capture the origin of their evolution. Hence, in the following sections, we discuss the potential impact of all results gathered from *in-situ* diffraction to explain the mechanical response of the micro-wires, starting from elementary deformation mechanisms up to microstructure and architecture contributions.

### Elementary deformation mechanisms

Before entering into the detailed discussion on deformation mechanisms, it should be recalled that previous studies on the Ni micro-wires have shown that grain growth does not occur during their tensile deformation^49^. The only region that sees significant grain size changes is the necking region where grain subdivision was observed. Here, the volume of necking region is negligible compared to the entire probed volume during *in-situ* tests. Hence, this grain subdivision does not induce noticeable peak broadening and it can reasonably be considered that all FWHM changes arise solely from modifications of the micro-strains landscape within grains (incl. dislocations).

Strain rate sensitivity in Ni is grain-size dependent. Study on nickel with microstructure made of NC, UFG or coarse grains (CG) suggested a strain rate dependency of flow stress only in NC regime:^66^ strain rate sensitivity index ‘m’ of Ni ranges from 0.02 to 0.04 for NC and tends to zero for CG and UFG. The strain rate dependency index for the current Ni microwires is 0.005 (presented in Supplementary results); hence, this very low value agrees well with literature data on bulk coarse grain Ni. Moreover, the low strain rate sensitivity index measured from strain rate jump tests indicates that plasticity proceeds via dislocation-based mechanisms rather than grain boundary sliding mechanisms.

This is further complemented by the stress-drop tests carried out on AA100 wires and detailed in Supplementary results. In the case of NC nickel^47^, four distinct regimes have been identified based on the evolution of inelastic strains and FWHM at different stress drop ratios. For large relative stress reduction ratio R (or small stress drops), where the applied stress is kept well above the internal stress, dislocations will continue to nucleate and/or propagate, causing a forward strain with positive changes in FWHM. For smallest relative stress reduction ratio R (large stress drops), a negative strain and continued reduction of FWHM are seen as a result of backward motion of dislocations due to high internal stresses. For intermediate relative stress reduction ratios, different cases are observed: a) positive strain accompanied by negative evolution of FWHM, b) transient decrease of inelastic strain followed by an increase accompanied by continuous decrease of FWHM. All the regimes characterized by a decrease of FWHM are associated with mechanisms involving grain boundary accommodation which result in relaxation of internal stresses. The stress drop tests carried out here on nickel microwires indicate that the inelastic strain always remains positive (for all stress drop ratios) with immediate reduction of FWHM that continues to diminish until the end of the creep period. The continued reduction of FWHM suggests that inhomogeneous strains decrease via grain boundary mediated processes that reduce the internal stresses in the plastic regime. A very low measured strain rate sensitivity index confirms that the Ni microwires deform only by dislocation-based mechanisms (and not by some sort of grain boundary sliding). Hence, the observed increase of inelastic strain, accompanied by reduction of FWHM can be attributed to a combination of movements of dislocations and their absorption at grain boundary, resulting in a sort of “cleaning-up” process of the grain interior with respect to dislocation content. *In-situ* monotonous and cyclic tensile test indicate a decrease of FWHM in the micro-plastic region: we attribute such decrease in FWHM to a similar “cleaning-up” process. This clean-up process can also be attributed as the primary mechanism responsible for the decrease of FWHM upon loading in the regime I and α from the monotonous and cyclic tensile test. Such a mechanism is not surprising in microstructure that has been obtained by severe drawing: lot of dislocation segments are bowing out of grain boundaries and are rather unstable towards applied stress. Similar behavior has been already observed in SPD-processed metallic wires^62^.

### Deformation of the polycrystalline aggregate in nickel microwires

The behavior of <111> grain family was observed to be rather similar in all wires: this can be easily understood as the majority of <111> grains are located in the center of the microwires (the core region). As the wire diameter is reduced, the macroscopic behavior of the wire becomes majorly governed by the behavior of <111> grains.

Plastic deformation in CG crystalline metals usually occurs by crystallographic slip, along {111} <110> slip systems in the case of FCC materials like Ni. According to the Schmid law, during a tensile test, <100> oriented single crystal (whose Schmid factor is equal to 0.408) should yield before <111> oriented single crystal (Schmid factor equal to 0.272). Moreover, directional Young’s modulus in nickel is the highest for <111> oriented grains (i.e. 288 GPa) and the lowest for <100> oriented grains (i.e. 131 GPa).

In the Ni microwires under study, all the grains are highly aligned and elongated along the tensile axis with two dominant orientations, <111> and <100>. These two major grain families can be seen as the two ‘phases’ of a unidirectional composite material where each ‘phase’ exhibits different elastic and plastic properties. Under iso-stress condition which is assumed within the Sachs model, yielding of <100> grains would occur first because of their high Schmid factor. Conversely, <111> grains would yield first in case of iso-strain condition (Taylor model) due to higher Young’s modulus. Experimentally, it was observed in the present study that <111> grains yield first. This suggests that the polycrystalline aggregate behaves more like a unidirectional composite in iso-strain mode, with the yield stress of the microwire being controlled by the volume fraction of <111> grains due to high Young’s modulus.

If we follow the evolution of lattice strain with respect to applied stress from Fig. 7(a), it can be seen that the <111> oriented grains plastify first (indicated by the sub regime II’, which marks the microstructural changes in <111> grains); this is followed by an effective load transfer towards the <100> oriented grains in the case of AA100 wire, which deteriorates with decreasing diameter. Under higher stress, plasticity sets in <100> oriented grains (indicated by the sub regime II″, which marks the microstructural changes in <100> grains. With the reduction of shell volume fraction by electropolishing, the volume fraction of <100> grains is also reduced. This result in a microwire with high overall volume fraction of <111> grains, leading to a less effective global load transfer.

The load transfer between different grain families can be seen as a mechanism for release of intergranular stresses between neighboring grains (represented by the changes in FWHM), thereby delaying the onset of strain localization. The volume fraction and spatial spread of <100> oriented grains in the shell of the microwire seems to affect its ductility, whereas the heterogeneous shell in the AA100 wire seems to control the effectiveness of load transfer (resulting in higher ductility). As a result of shell removal by electropolishing (EP40 microwires) such mechanisms are not operative anymore and, therefore, leads to a reduced ductility.

Load-unload tests were performed to characterize the evolution of microstructure by following the evolution of peak profile in the elastic, micro-plastic and macro-plastic regimes. Three regimes were defined based on the behavior of FWHM obtained from monotonous tensile (regimes I, II and III) and cyclic tensile test (regime α, β and γ). A comparison of these regimes for the AA100 and EP40 wires is provided in Table 2 and Table 3.

**Table 2:** Definition of regimes for AA100 wire during monotonous and load-unload tests.

	Reflections	Regimes			
		I	II′	II″	III
Monotonous	(111)	Elasticity	Micro-plasticity		Macro-plasticity
	(200)	Elasticity		Micro-plasticity	Macro-plasticity
Load-unload cycle	(111)	α	β		γ
	(200)	α		β	γ

**Table 3:** Definition of regimes for EP40 wire during monotonous and load-unload tests.

	Reflections	Regimes			
		I	II′	II″	III
Monotonous	(111)	Elasticity	Absent	Micro-plasticity	Absent
	(200)	Elasticity		Micro-plasticity	Absent
Load-unload cycle	(111)	α	Absent	β	Absent
	(200)	α		β	Absent

The regime I and III can be clearly identified based on the behavior of FWHM: regime I is associated with elastic behavior, while regime III is associated with macro-plasticity (i.e. dislocation storage in all grains). Regime II has two sub-regimes i.e. sub-regime II′ marks the micro-plasticity in <111> grains and sub-regime II″ marks micro-plasticity in <100> grains. In the case of EP40 microwires, regime II is only composed of sub-regime II″, indicating that micro-plasticity of all grain families is imposed by the presence of higher volume fraction of <111> textured grains. These features are also reflected in the results from load-unload tests indicating that changes in deformation sequence occur as a result of the presence/absence of shell region.

Regime α is associated to the “cleaning up” process taking place below the macroscopic yield stress, where the residual dislocations generated by the cold-drawing process are being absorbed, causing a net decrease in FWHM for both (111) and (200) reflections (refer to Fig. 8(d)). Regime γ marks the homogeneous plastic deformation state represented by net increase in FWHM, indicating the activation of new sources and storage of dislocations by interaction within grain volume. Such a regime of homogeneous plastic deformation is not reached in the case of EP40 wire as can be seen from Fig. 9.

### Effect of microstructure (grain size, shape and arrangement)

Previous study on SPD nickel samples suggests that elongated grains have no considerable effect on the tensile strength and elongation to failure at room temperature^69^. The strength is rather governed by the average grain size perpendicular to the tensile axis. In the present study also, the influence of grain aspect ratio on the deformation mechanism seems rather limited, although this influence can be indirectly seen with respect to iso-strain mode resulting in early yielding in <111> grains. Other studies have shown that having a multimodal grain distribution or having a gradient in grain size from surface to the core can result in material with a more optimum combination of strength and ductility^44,45,70,71^. In the current study, the variation of grain size along the cross-section of the microwire is in the range of 400 nm to micrometer with no strong grain size gradient. However, a strong gradient in texture resulting in a core-shell architecture across the diameter of these microwires was revealed; this core-shell architecture seems to actually have the strongest effect on the mechanical properties.

### Tailoring properties via microstructure architecture

Commercially procured 100 µm nickel microwires exhibiting core-shell architecture showed an increase in tensile strength with reduction in diameter by electropolishing (extreme case being the complete removal of shell below 40 µm). This strengthening is attributed to the core-shell type of microstructure or “architecture effect” where the core consists mainly of <111> textured grains. The strength of the microwire is thus controlled by the volume fraction of the <111> texture. The presence of heterogeneously textured shell in the 100 µm wire provides additional release of internal stresses by grain-to-grain load transfer thereby delaying strain localization and consequently, increasing ductility. It can be anticipated that higher ductility without compromising too much on strength in AA100 wires could be attained by having a heterogeneous core with dual <100> and <111> fiber texture, enabling the load transfer and release of intergranular stresses, thereby increasing the failure strain of the microwire.

Hence, coexistence of <111> and <100> grains in addition to microstructure heterogeneity (core-shell architecture) can probably be considered as the tuning parameters for optimizing the combination of strength and ductility. In other words, microstructure engineering on micro-texture components (single- or dual-texture) and their spatial spread (homogenous or architectured) may be used as design guidelines for obtaining optimal microstructure in accordance with desired set of properties. Crystal plasticity finite element (CPFE) simulations are currently under progress to assess the individual contributions of these parameters.

## Conclusion

In the present study, the effect of diameter reduction on the mechanical properties of cold-drawn nickel microwires has been analyzed. The studied microwires exhibit duplex fiber texture (<100>, <111>) core-shell architecture: core with <111> oriented grains and shell with <111> and <100> oriented grains. The intermediate diameters with reduced shell were obtained from the initial cold-drawn microwires via electropolishing. An increase in strength with reduction in failure strain is observed upon reduction of wire diameter by this technique. The tensile strength ranged from 885 MPa with an elongation to failure of 1.2% for 100 µm wires to a maximum tensile strength of 1110 MPa with an elongation to failure of 0.5% for electropolished 40 µm wires. Complex *in-situ* tests (monotonous, cyclic tensile, strain rate jumps and stress drop) were successfully performed at room temperature on these microwires (for the first time) under high-energy X-ray beams to gain insights into deformation mechanisms. The following conclusions were drawn:

1. The low strain rate sensitivity index and the forward inelastic strain over different stress drop ratios indicate that the deformation in these microwires takes place by dislocation-based mechanisms. Stress drop tests show that grain boundaries play a significant role in the first stages of plastic deformation (dislocations sinks).
2. The evolution of lattice strains and FWHM with respect to applied stress indicate successive yielding of grain families in the microwire with <111> oriented grains reaching plasticity first (sub-regime II′). Thereafter, plasticity sets in <100> oriented grains resulting from the effective load transfer between <111> and <100> oriented grains (sub-regime II″). A significant load transfer between <111> and <100> oriented grains is seen in the case of 100 µm wires, which diminishes with the reduction of wire diameter.
3. A direct influence of microwire’s architecture on the strength and ductility was observed, which establishes that the effective load transfer within the microwire, as a result of specific microstructure architecture, provides a feasible way to tune the mechanical properties (strength and ductility). This architecture effect should play important role in developing microstructures with superior mechanical properties.

The insights from the present study may contribute meaningfully to the overall study and understanding of the role of texture and its arrangement, the architecture, with respect to mechanical properties in FCC metals.

## Electronic supplementary material

Supplementary information

## Data Availability

The datasets generated and analyzed during the current study are available from the corresponding author on reasonable request.
