# Supplementary material for: Revealing the role of microstructure architecture on strength and ductility of Ni microwires by in-situ synchrotron X-ray diffraction
Source: Sci Rep. 2019 Jan 11;9:79. doi: 10.1038/s41598-018-36472-3 (PMC6329826; doi:10.1038/s41598-018-36472-3)
Supplement: Supplementary file 1 — Supplementary information [file 41598_2018_36472_MOESM1_ESM.docx]

**Supplementary materials to article “Revealing the role of microstructure architecture on strength and ductility of Ni microwires by *in-situ* synchrotron X-Ray diffraction”**

**Ravi raj purohit PURUSHOTTAM RAJ PUROHIT^1^, Abhinav ARYA^2^, Girish BOJJAWAR^2^, Maxime PELERIN^3^, Steven VAN PETEGEM^4^, Henry PROUDHON^3^, Soham MUKHERJEE^1^, Céline GERARD^1^, Loïc SIGNOR^1^, Cristian MOCUTA^5^, Nicola CASATI^6^, Satyam SUWAS^2^, Atul H. CHOKSHI^2^, Ludovic THILLY^1*^**

*^1^Institut Pprime, CNRS – ENSMA – Université de Poitiers, Département Physique et Mécanique des Matériaux, 86961 Futuroscope, France*

*^2^Department of Materials Engineering, Indian Institute of Science, Bangalore 560 012, India*

*^3^MINES Paris Tech, Centre des Matériaux, CNRS UMR 7633, BP 87 91003 Evry Cedex, France*

*^4^Photons for Engineering and Manufacturing, Swiss Light Source, Paul Scherrer Institute, Villigen, Switzerland*

*^5^Synchrotron SOLEIL, L’orme des Merisiers, Saint Aubin – BP 48, Gif-sur-Yvette, 91192, France*

*^6^Laboratory for Synchrotron Radiation - Condensed Matter (LSC), Swiss Light Source, Paul Scherrer Institute, Switzerland*

**ludovic.thilly@univ-poitiers.fr*

**SUPPLEMENTARY METHODS**

***In-situ* experimental setup and diffraction geometry**

The microwire was glued in the V-shape groove of one specific holder using a cyanoacrylate-based glue (‘Loctite’), with the assistance of an optical microscope and high magnification camera to achieve a good alignment within the groove (refer to Supplementary Figure S1). Mounted samples were further imaged at sufficient magnification with SEM for checking wire alignment.

The diffraction geometry corresponding to the transverse and axial (hkl) reflections are illustrated in Supplementary Figure S2. The blue vector corresponds to the diffraction vector associated with planes perpendicular to wire axis (labelled axial (hkl) reflections) and the red vector corresponds to the diffraction vector associated with the planes parallel to wire axis (labelled transverse (hkl) reflections).

At the DiffAbs beamline, only a part of the Debye-Scherrer rings was recorded using the area detector (XPAD S140 [1]). The acquisition time of the detector at DiffAbs beamline was 1 second per pattern for AA100 wire and 3 seconds per pattern for EP40 wire. The recorded Debye-Scherrer rings here correspond to the reflections associated to planes perpendicular to the wire axis and the consequently calculated lattice strains correspond to axial strains. The advantage of axial geometry is that each axial reflection corresponds to the respective texture component of the grain families (refer to blue planes in Supplementary Figure S2).

At the MS beamline, a part of the Debye-Scherrer rings was also recorded using the area detectors (Pilatus 6M [2]). The acquisition time used was 10 seconds per pattern for the AA100 wire and 30 seconds per pattern for the EP40 wire. The recorded Debye-Scherrer rings correspond to the reflections associated to planes perpendicular to the wire axis. Along with the axial reflections, the transverse (hkl) component of the textured grains was also simultaneously recorded for all the type of tests with a 1D microstrip detector (Mythen [3]) having a 2θ range of 120° and an angular resolution of 0.0039°. In this geometry, the observed peaks correspond to the transverse reflection (refer to red planes in Supplementary Figure S2).

**Stress-drop test**

All stress drop tests were carried out in the macro-plastic region defined by the 0.2% strain criterion which is met at a stress of around 850 MPa for AA100 wires. One example of the stress drop test is shown in Supplementary Figure S3 (each data point corresponds to the recorded XRD patterns). The stress drop tests were conducted by straining the microwires up to a pre-defined stress (σ_0_) at a constant strain rate of $3\times{10}^{-5} s^{-1}$ and then dropping the stress suddenly to a new lower level (σ_r_) and staying at the new level for a creep period of 60 minutes. After this period, constant strain rate straining can be resumed up to the pre-defined stress, if necessary. In terms of analysis, only the evolution of FWHM and the inelastic strain during the 60min creep period is of interest. The inelastic strain is calculated from the macroscopic stress-strain curve by subtracting the contribution of elastic strain from total strain using the following relation:

| $\varepsilon_{inelastic}= \varepsilon_{total}- \frac{\sigma}{E_{app}}$ | (1) |
| --- | --- |

where E_app_ is the apparent elastic modulus which includes the contribution of the MTM. The relative stress reduction ratio is defined as$R ={\sigma_{r}}/{\sigma_{0}}$: for a large stress reduction, R is very small.

Stress drop tests allow studying the deformation mechanism responsible for the changes in FWHM in the macro-plastic regime by suppressing dislocation slip. A more elaborate study of such stress drop tests on electrodeposited NC Ni can be found in reference [4].

**Strain rate jump tests**

Strain rate jump tests were carried out at MS beamline on the AA100 wires. As shown in Supplementary Figure S4, a method of sudden increase and decrease of strain rate in the macro-plastic regime is employed to calculate the strain rate sensitivity index ‘m’. The strain rate was sequentially modified to values between $5\times{10}^{-5} s^{-1}$ and$1\times{10}^{-3} s^{-1}$, and sufficient time in-between to avoid any transient effect. The strain rate sensitivity index is calculated with the following equation:

| $m= \frac{ln({\sigma_{A}}/{\sigma_{B}})}{ln({\dot{\varepsilon}_{B}}/{\dot{\varepsilon}_{A}})}$ | (2) |
| --- | --- |

where σ_A_ and σ_B_ are the respective stress level at strain rates $\dot{\varepsilon}_{A}$ and$\dot{\varepsilon}_{B}$. The analysis of the strain rate sensitivity index provides information on the nature of deformation. Higher values of m (>0.3) are considered as footprint of superplastic behavior. A strain rate sensitivity index of nearly zero indicates rate-independent plastic flow. A detailed study on strain rate sensitivity for micro- (>1 µm), ultrafine (100-1000 nm) and nano-grain (<100 nm) Ni can be found in references [5, 6].

**SUPPLEMENTARY RESULTS**

**Stress drops**

*In-situ* stress drop tests have been carried out on AA100 microwires with stress drop ratio ranging between 0.48 and 0.90. Supplementary Figure S5 shows the evolution of inelastic strain and FWHM of (111) and (200) reflections during a creep period of 60 minutes. For all stress drop ratios, we observe a continuous increase in inelastic strain with creep time. Also, an immediate decrease in FWHM for both the grain families is seen at the beginning of creep for all stress drop ratios. The FWHM still continues to decrease over time but at a relatively slow rate.

**Strain rate sensitivity**

The strain rate sensitivity of Ni microwires was probed by carrying out a series of increase and decrease of strain rate during the continuous tensile test (see Supplementary Figure S4). Hence the strain rate jumps are carried out in the macro-plastic regime of AA100 wire, which is possible here due to the presence of an extended zero hardening regime. The strain rate sensitivity index calculated from the stress strain curve in Supplementary Figure S4 ranges from: m(a,b)= 0.00502, m(b,c)=0.00638, m(c,d)=0.0062, m(d,e)=0.00491, averaged to 0.0056 ± 0.00077.

**SUPPLEMENTARY REFERENCES**

[1] Boudet, N. et al, XPAD: a hybrid pixel detector for X-ray diffraction and diffusion. Nucl. Instrum. Methods Phys. Res, 510, 41-44 (2003).

[2] Rajendran, C. et al, Radiation damage in room-temperature data acquisition with the PILATUS 6M pixel detector. J. Synchrotron Radiat, 18, 318-328 (2011).

[3] Bergamaschi, A. et al, The MYTHEN detector for X-ray powder diffraction experiments at the Swiss Light Source. J. Synchrotron Radiat, 17, 653-668 (2010).

[4] Sun, Z. et al. Dynamic recovery in nanocrystalline Ni. Acta Mater., 91, 91-100 (2015).

[5] Schwaiger, R. et al, Some critical experiments on the strain-rate sensitivity of nanocrystalline nickel. Acta Mater., 51, 5159-5172 (2003).

[6] Van Petegem, S., Zimmermann, J. & Van Swygenhoven, H. Microstructure and deformation mechanisms in nanocrystalline Ni–Fe. Part II. In situ testing during X-ray diffraction. Acta Mater., 61, 5846-5856 (2013).


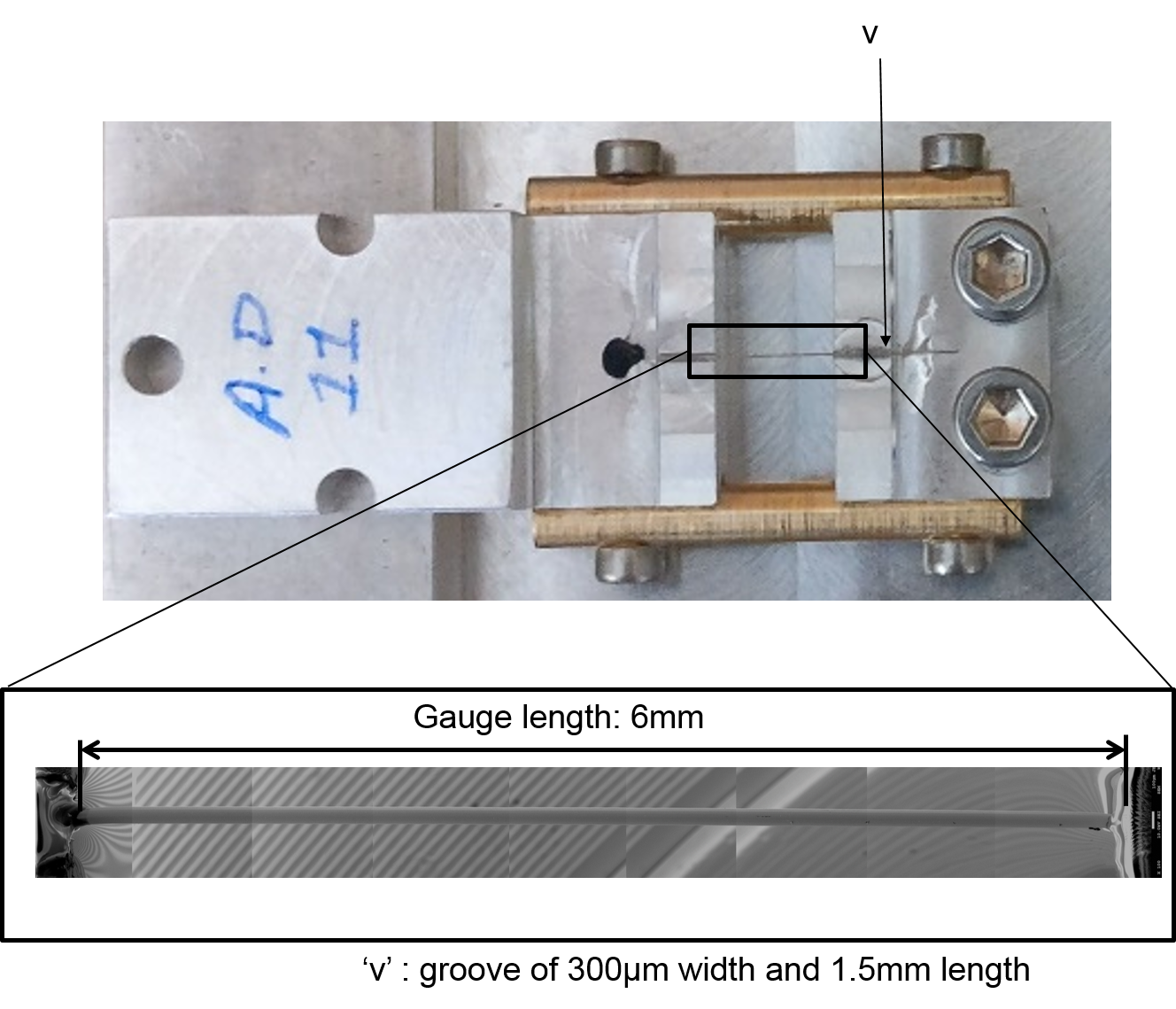


Supplementary Figure S1. Microwire holder (‘v’: V-shape groove with 300 µm width and 1.5mm length). Two lateral bars are in place to keep the sample from moving during handling. The inset shows the stitched SEM images of the wire. The left end of the wire is glued with a patch of conductive colloidal graphite to avoid charge build-up during SEM.


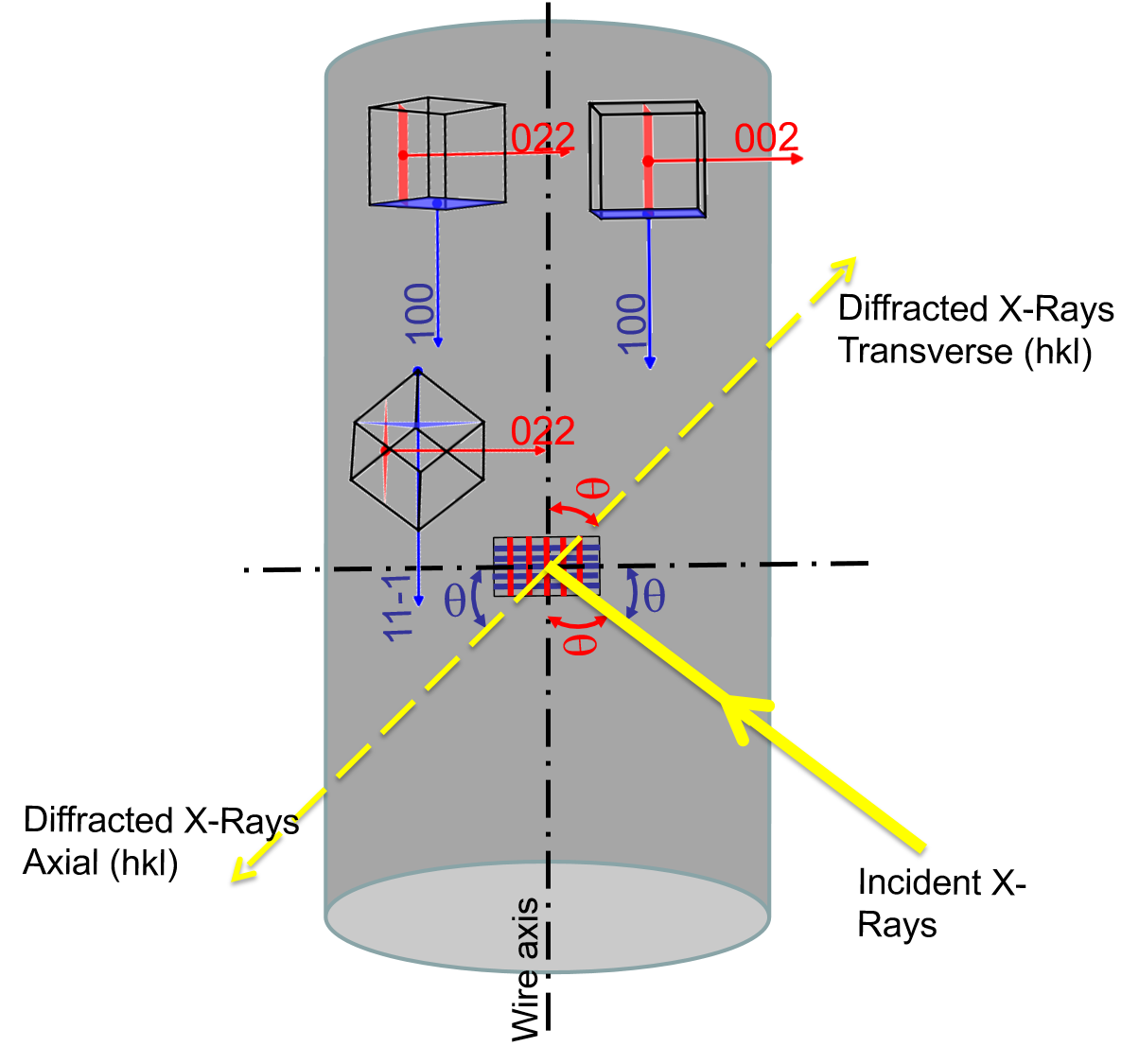


Supplementary Figure S2. X-Ray diffraction of nickel microwire with major texture components. Both axial and transverse (hkl) reflections were recorded at MS beamline. At the DiffAbs beamline, only the axial (hkl) reflections were recorded.


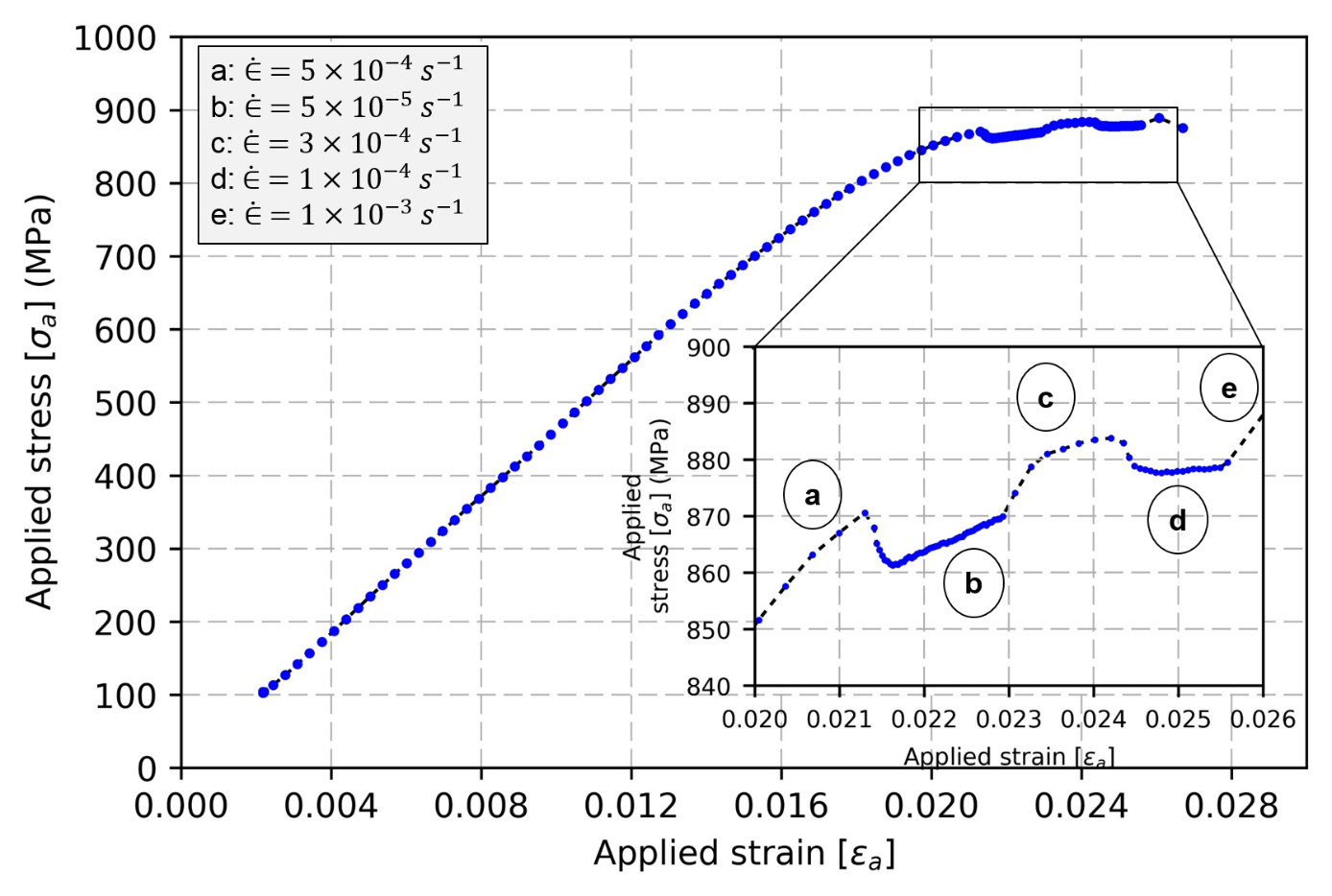


Supplementary Figure S3. Stress drop tests for one AA100 microwire. Top inset shows stress drop test for one stress ratio defined by a pre-defined stress $\sigma_{0}$and the current unloaded stress$\sigma_{r}$. Bottom inset shows the evolution of macroscopic applied strain during the creep period.


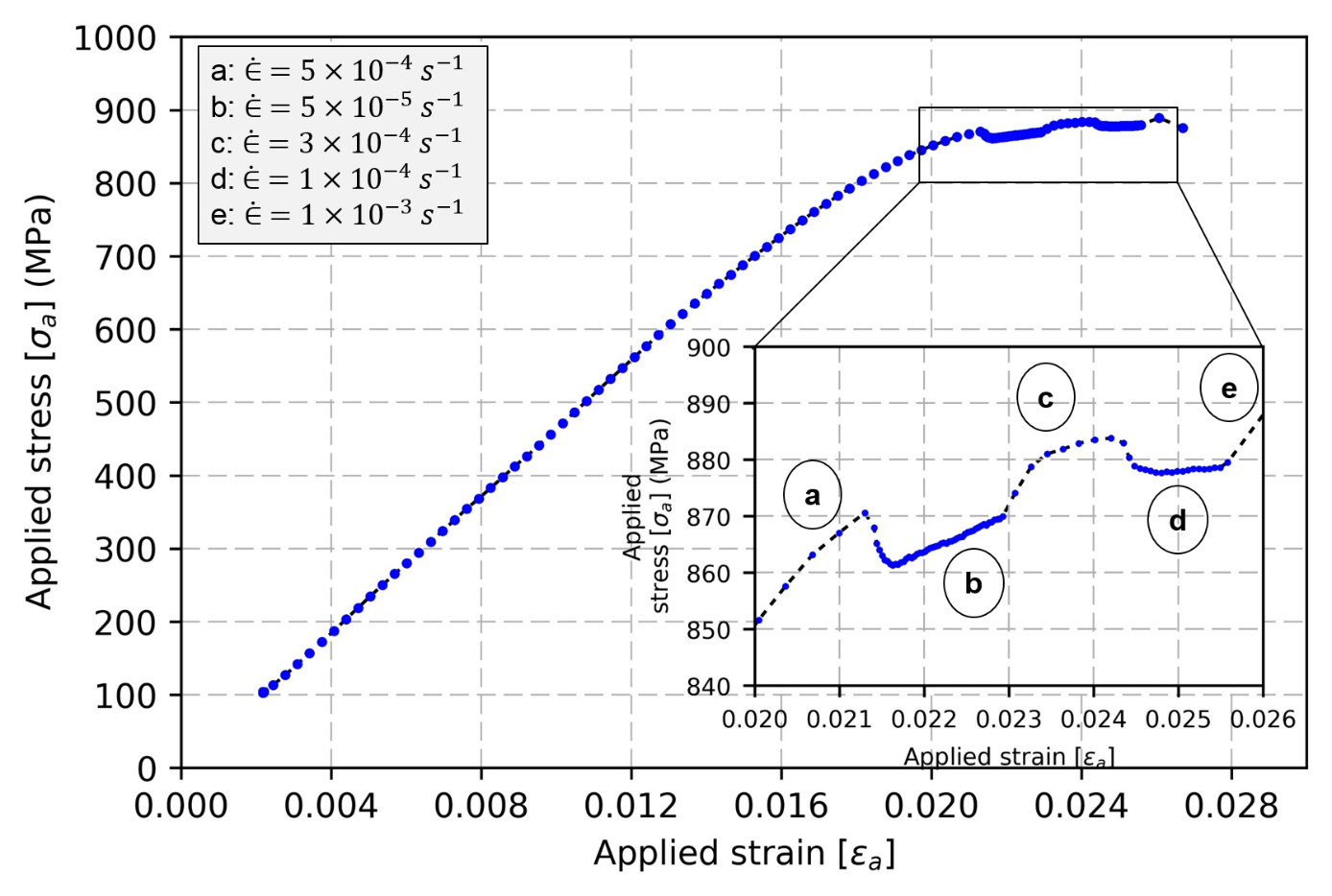


Supplementary Figure S4. Continuous stress-strain curve for AA100 microwire with different strain rates in the macro-plastic regime. Inset shows the magnified section the curve where the effect of strain rate changes can be seen.


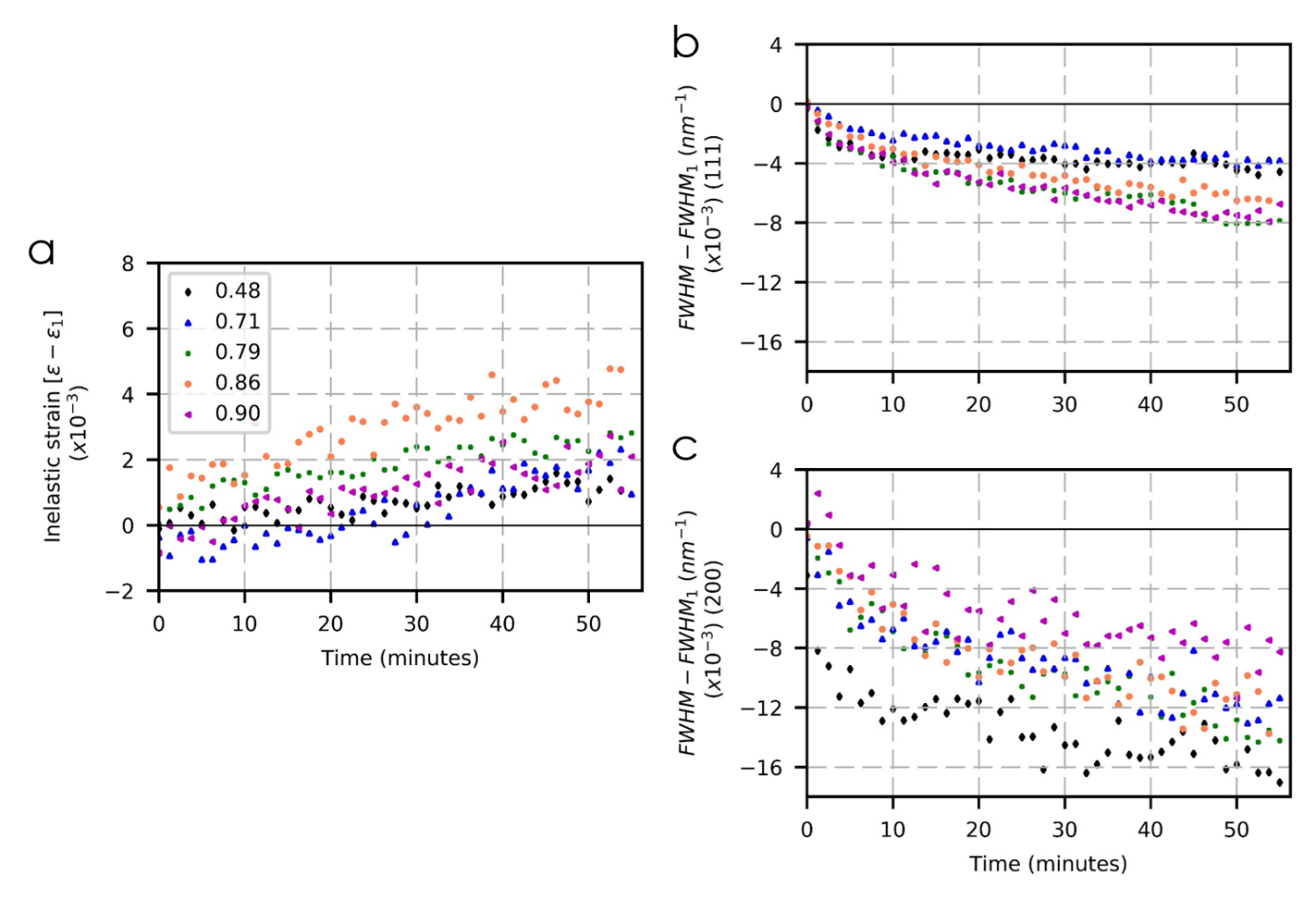


Supplementary Figure S5. Stress drop tests on AA100 microwires for various stress drop ratios. a: inelastic strain versus time, b: evolution of FWHM for the creep period of 60 minutes for <111> textured grains, c: evolution of FWHM for the creep period of 60 minutes for <100> textured grains.
